# Supplementary material for: Delayformer: Spatiotemporal Transformation for Predicting High‐Dimensional Dynamics
Source: Adv Sci (Weinh). 2025 Nov 21;13(3):e14671. doi: 10.1002/advs.202514671 (PMC12806438; doi:10.1002/advs.202514671)
Supplement: Supplementary file 1 — Supporting Information [file ADVS-13-e14671-s001.pdf]

Supplementary Information

for

Delayformer: spatiotemporal transformation for predicting high-dimensional dynamics

Supplementary Note 1. Implementation Details

1.1 Datasets in long-term multivariate time series forecasting

We conducted our experiments on 8 most popular real-world datasets for multivariate time series forecasting and two synthetic datasets. **ETT** (Electricity Transformer Temperature) datasets are collected from electricity transformers at two locations and labeled with 1 and 2, covering 7 variables. ETT datasets contain 4 datasets: **ETTh1**, **ETTh2**, **ETTm1**, and **ETTm2**, where h and m represent two sampling intervals: 1 hour (h) and 15 minutes (m). **Weather** dataset collects 21 meteorological indicators every 10 minutes from Germany (e.g., humidity, air temperature) in 2020. **Solar-Energy** dataset describes 137 solar power plants every 10 minutes in 2006. **Electricity** dataset records the electricity consumption every 1 hour from 321 customers. **Traffic** dataset contains road occupancy rates from 862 sensors in San Francisco. The two synthetic datasets include a 30-dimensional time-invariant Lorenz system and a 30-dimensional time-varying Lorenz system, which are illustrated in Methods.

1.2 Algorithm Implementation Flow of Delayformer

Algorithm 1. Generic structure of Delayformer

Input: input time sequence $\mathbf{X} \in \mathbb{R}^{N \times W}$ with N dimension and length W; delay embedding dimension L; patching size (p_1, p_2); transformer encoder number n

1. **for** $k = 1, \dots, N$ **do**
2. Construct Hankel matrix $\mathbf{Y}_k^{Known}$ for k-th variable from time series data $\{x_k^t\}_{t=1}^{W}$
3. Slice $\mathbf{Y}_k^{Known}$ to patches P_k with size of (p_1, p_2)
4. $H_k^0 = \mathrm{MLP}(flatten(P_k))$
5. Add position encoding $H_k^0 = H_k^0 + PE$
6. **for** $j = 1, \dots, n$ **do**
7. $H_k^j = \text{Transformer Encoder Block}(H_k^{j-1})$
8. **End**

9. $\widehat{\mathbf{X}_k}^{Pred} = \mathbf{f}_k(H_k^n)$

10. **End**

Output: m step prediction $\widehat{\mathbf{X}}^{Pred}$

1.3 Selection of hyperparameters in Delayformer

Embedding dimension (L): This parameter determines the shape of the Hankel matrix. While theoretical methods exist for their calculation, they can sometimes yield extreme values. A practical approach is to set L to approximately half the input sequence length. This makes the resulting Hankel matrix as close to a square as possible, which is often beneficial for vision-based backbones like ViT.

Patching size (p_1, p_2): The choice of patching size is a trade-off between capturing fine-grained local features and computational efficiency. Smaller patches increase the number of tokens and the computational load, while larger patches may miss important local patterns. The values listed in Supplementary Table 1 provide effective starting points that balance this trade-off.

1.4 Baseline used in the main text

In Table 1 and Fig. 2-4, we selected several baselines, including transformer-based models: iTransformer, PatchTST[1], Crossformer[2], FEDformer[3], Stationary[4] and Autoformer[5], TCN-based models: SCINet[5] and TimesNet[6] and linear models: RLinear[7], DLinear[8] and TiDE. The implementation of the baselines is from https://github.com/thuml/Time-Series-Library. To make the results in each model comparable, the input sequence length was all set to be 96.

Supplementary Note 2. Real-world Meteorological Dataset

Datasets shown in Fig. 4 include three public meteorological datasets: **wind speed**, **solar irradiance**, and **ozone level**. **Wind speed** dataset records the wind speed time series data every 10 minutes between 2010 and 2012 from 155 wind stations in Wakkanai, Japan. **Solar irradiance** dataset contains the solar irradiance strength every 10 minutes between 2010 and 2012 from 155 observation stations in Wakkanai, Japan. **Ozone level** dataset collects 72 air quality features every 1 hour from 1998 to 2004 in Houston, Galveston and Brazoria area.

Supplementary Note 3. Hyperparameter and Environment Used in Long-Term Forecasting Benchmark Dataset

All the experiments used in the main text were implemented with PyTorch on a single NVIDIA A100 40GB GPU. Adam optimizer[9], Layer Normalization[10], and L2-loss were used in all experiments of Delayformer. Delayformer is an encoder-only structure, and each transformer encoder consists of two multilayer perceptron layers and one multi-head attention layer. The dropout probability and total training epoch were set as 0.1 and 10 for all cases. The detail hyperparameters in the experiments in shown in Supplementary Table 1.

Supplementary Note 4. The Demo of Pretrained Delayformer

As large models rapidly approach dominance in a variety of domains, we explored the potential of Delayformer by conducting experiments on two cases, described in the main text. We based our approach on the parameter scale of current time series foundation models, transforming all data into univariate sequences and constructing a 10-layer Delayformer architecture. The Delayformer model was pretrained and subsequently fine-tuned using varying ratios of available training data. The prediction length and input length were both set to 96, with the number of attention heads fixed at 12. The embedding dimension of Delayformer was set to 49, and the parameters (p_1, p_2) were defined as $(24, 7)$. The model was trained for 15 epochs during pretraining and 10 epochs during fine-tuning. The full results are shown in Supplementary Table 4.

Supplementary Note 5. Ablation of Shared Encoder and Independent Decoder Design

To validate the architectural choice of adopting a shared encoder with independent linear decoders in Delayformer, we conducted an ablation study comparing two variants: (i) shared encoder + shared decoder, (ii) shared encoder + independent decoders (Delayformer). Results of (ii) are shown in Table 1 in the main text.

This design explicitly combines the robustness of channel-independent feature extraction with the precision of channel-specific prediction. The shared encoder focuses on capturing universal temporal dynamics that are stable across variables, while lightweight channel-specific decoders adapt to heterogeneous scales and units of different variables.

We evaluated the models on two representative datasets with different levels of heterogeneity: ETTh2 (homogeneous) and Weather (heterogeneous). As shown in Supplementary Table 5, Delayformer (shared encoder + independent decoders) consistently outperformed the shared-decoder variant on the heterogeneous Weather dataset and the homogeneous ETTh2 dataset. These results confirm the necessity of independent decoders when handling heterogeneous real-world data.

Supplementary Note 6. Comparison between ViT and CNN Backbones

Delayformer provides an effective approach for modeling time series data as images. Convolutional neural networks (CNNs), being a fundamental architecture for feature mapping in images, serve as a natural candidate for comparison with the original ViT backbone in our model. To evaluate the effectiveness of CNNs as an alternative vision backbone, we implemented Delayformer with CNN-based encoders and compared its performance with the original ViT-based design under identical experimental settings.

The results are summarized in Supplementary Table 6 and Table 1. On both ETTh2 (hourly resolution) and Weather (10-minute resolution) datasets, the ViT backbone consistently outperformed the CNN variant. We attribute this to the ViT's capability of modeling both short- and long-term temporal dependencies via global self-attention, whereas CNNs are limited by their local receptive fields and thus less effective at capturing long-range dynamics.

These results highlight the advantage of ViT in handling complex temporal structures, particularly in high-frequency datasets such as Weather, where long-range dependencies are critical.

Supplementary Note 7. Impact of Input Sequence Length on Model Performance

To assess the robustness and scalability of Delayformer, we investigated its performance under varying input sequence lengths. We conducted experiments on the ETTh2 dataset with input lengths of 48, 96, 128, 256, and 512. For all experiments, the prediction horizon was kept fixed at 96 steps to ensure a fair comparison. Other hyperparameters remained consistent with those reported in Supplementary Table 1 except L and (p_1, p_2) (change as input length changes) to isolate the effect of input length.

The results, visualized in Supplementary Fig. 16, demonstrate a clear trend. As the input sequence length increases from 48 to 128, the model's performance, measured by Mean Squared Error (MSE) and Mean Absolute Error (MAE), steadily improves. This is expected, as longer input sequences provide more historical context, enabling the model to better capture complex temporal patterns and dependencies within the data.

However, we observed a slight degradation in performance when the input length was further extended to 256 and 512. This suggests a trade-off: while sufficient historical data is beneficial, excessively long sequences may introduce outdated patterns or noise that can interfere with the model's ability to focus on the most salient recent dynamics for short-term forecasting.

Overall, Delayformer demonstrates strong and robust performance across a wide range of input lengths. The results confirm that the model can effectively leverage historical information, and its performance is not overly sensitive to the precise choice of input length within a reasonable range (e.g., 96 to 128), highlighting its suitability for diverse practical applications.

Supplementary Note 8. Robustness Analysis under Varying Input Lengths and Prediction Horizons on the Lorenz System

To further evaluate the robustness and generalizability of Delayformer on chaotic systems, we conducted additional experiments on the time-invariant, noise-free 30-dimensional coupled Lorenz system by systematically varying the input sequence length and the prediction horizon. These experiments were designed to address the fundamental limits of predictability in chaotic dynamics and to test Delayformer's behavior beyond the standard 96-in/96-out benchmark used in the main text (Fig. 2).

Two sets of experiments were performed:

1. Varying Input Length (Prediction Horizon Fixed at 96):

We tested input lengths of 48, 96, 128, and 256. Contrary to intuition, the shortest input (48 steps) yielded the best forecasting accuracy. This phenomenon can be explained by the sensitive dependence on initial conditions inherent to chaotic systems. While longer inputs theoretically provide more information for state-space reconstruction via delay embedding, they also accumulate numerical errors and amplify trajectory divergence over time. In practice, for a system with a short Lyapunov time (i.e., rapid loss of predictability), excessively long historical sequences introduce more chaotic noise than useful dynamical structure. Thus, an input length of 48 strikes an optimal balance between sufficient state reconstruction and minimal error propagation in this specific setting.

2. Varying Prediction Horizon (Input Length Fixed at 96):

We tested prediction horizons of 50, 96, 150, and 200 steps. As expected from dynamical systems theory, forecast error increases monotonically with horizon length due to the positive Lyapunov exponents of the Lorenz attractor. This exponential divergence is an intrinsic property of chaos and is independent of the forecasting model. The consistent degradation in performance (measured by both MSE and MAE) validates that Delayformer operates near the theoretical predictability limit of the system.

These results demonstrate that Delayformer not only achieves state-of-the-art performance under standard benchmark conditions but also exhibits behavior that aligns precisely with the theoretical expectations for chaotic forecasting. The full numerical results are reported in Supplementary Table 7.

Supplementary Tables

Supplementary Table 1. Hyperparameters in the Experiments in the Main Text

Dataset	Input Length	Predict Length	Layer Number	Head Number	Model Dim	FFN Dim	Batch Size	Learning Rate	Embedding Dim	(p_1, p_2)
ETTh1	96	96	3	12	256	512	128	0.0001	27	(5, 3)
		192	3	12	256	512	128	0.0001	27	(5, 3)
		336	3	12	128	1024	128	0.0001	49	(6, 7)
		720	3	12	128	2048	128	0.0001	49	(6, 7)
ETTh2	96	96	3	12	128	2048	128	0.0001	49	(6, 7)
		192	3	12	256	512	128	0.0001	49	(6, 7)
		336	3	12	256	512	128	0.0001	49	(6, 7)
		720	3	12	128	128	128	0.0001	27	(5, 3)
ETTm1	96	96	3	12	128	2048	128	0.0001	27	(5, 3)
		192	3	12	128	2048	128	0.0001	27	(5, 3)
		336	3	12	128	2048	128	0.0001	27	(5, 3)
		720	3	12	128	2048	128	0.0001	27	(5, 3)
ETTm2	96	96	3	12	128	128	128	0.0001	49	(6, 7)
		192	3	12	128	128	128	0.0001	49	(8, 7)
		336	3	12	128	128	128	0.0001	49	(4, 7)
		720	3	12	128	128	128	0.0001	49	(4, 7)
Weather	96	96	3	12	128	2048	64	0.0001	27	(5, 3)
		192	3	12	128	2048	64	0.0001	49	(6, 7)
		336	3	12	128	2048	64	0.0001	49	(6, 7)
		720	3	12	128	2048	64	0.0001	49	(6, 7)
Solar-Energy	96	96	2	8	512	512	128	0.0005	49	(24, 7)
		192	2	8	512	512	128	0.0005	49	(24, 7)
		336	2	8	512	512	128	0.0005	49	(24, 7)
		720	2	8	512	512	128	0.0005	49	(24, 7)
Electricity	96	96	2	12	512	512	32	0.0005	57	(10, 19)
		192	2	12	512	512	32	0.0005	57	(10, 19)
		336	2	12	512	512	32	0.0005	57	(10, 19)
		720	2	12	512	512	32	0.0005	57	(10, 19)
Traffic	96	96	2	8	512	512	16	0.001	49	(24, 7)
		192	2	8	512	512	16	0.001	49	(24, 7)
		336	2	8	512	512	8	0.001	49	(8, 49)
		720	2	8	256	512	4	0.001	49	(8, 49)
Wind-Speed	48	45	1	8	32	32	4	0.00001	24	(5, 6)
Solar-Irradiance	96	140	1	8	64	512	4	0.00001	49	(8, 7)

Ozone-level	24	25	1	8	32	32	4	0.00001	10	(5, 5)
Lorenz	96	96	3	8	128	2048	128	0.0001	49	(24, 7)

Supplementary Table 2. Long-Term Forecasting with Different Delayformer Hyperparameters in Fig. 5b

Predict length	(p1, p2)=(5, 3) m=12		(p1, p2)=(5, 3) m=27		(p1, p2)=(5, 3) m=42	
	MSE	MAE	MSE	MAE	MSE	MAE
96	0.161	0.209	0.161	0.210	0.161	0.209
192	0.208	0.252	0.208	0.252	0.208	0.252
336	0.264	0.293	0.266	0.294	0.265	0.294
720	0.346	0.346	0.348	0.347	0.345	0.345

Supplementary Table 3. Long-Term Forecasting with Different Delayformer Hyperparameters in Fig. 5c

Predict length	(p1, p2)=(3, 7) m=49		(p1, p2)=(6, 7) m=49		(p1, p2)=(8, 7) m=49		(p1, p2)=(12, 7) m=49	
	MSE	MAE	MSE	MAE	MSE	MAE	MSE	MAE
96	0.161	0.210	0.161	0.210	0.161	0.210	0.162	0.210
192	0.208	0.252	0.208	0.252	0.209	0.253	0.209	0.253
336	0.266	0.294	0.264	0.293	0.266	0.293	0.266	0.294
720	0.345	0.346	0.345	0.346	0.347	0.347	0.349	0.348

Supplementary Table 4. Results of the Demo of Pretrained Delayformer

Data %	ETTm2	
	MSE	MAE
zero-shot	0.139	0.254
1%	0.139	0.254
2%	0.137	0.252
5%	0.126	0.242
10%	0.127	0.245
20%	0.123	0.240
50%	0.122	0.235
100%	0.119	0.233

Data %	Weather	
	MSE	MAE
zero-shot	0.243	0.282
1%	0.196	0.239
2%	0.186	0.234
5%	0.188	0.237
10%	0.177	0.217
20%	0.188	0.188
50%	0.177	0.177
100%	0.175	0.175

Supplementary Table 5. Results of shared encoder + shared decoder structure on ETTh2 and Weather

Predict Length	ETTh2	
	MSE	MAE
96	0.294	0.343
192	0.389	0.400
336	0.431	0.440
720	0.438	0.452

Predict Length	Weather	
	MSE	MAE
96	0.162	0.208
192	0.210	0.251
336	0.268	0.292
720	0.345	0.343

Supplementary Table 6. Results of Delayformer (CNN-backbone) on ETTh2 and Weather

Predict Length	ETTh2	
	MSE	MAE
96	0.337	0.373
192	0.419	0.422
336	0.464	0.457
720	0.491	0.478

Predict Length	Weather	
	MSE	MAE
96	1.589	0.784
192	1.759	0.838
336	1.968	0.904
720	2.386	1.026

Supplementary Table 7. Forecasting Performance of Delayformer on the 30D Lorenz System under Varying Input Lengths and Prediction Horizons

Experiment Type	Input Length	Prediction Length	MSE	MAE
Varying Input	48	96	0.741	0.648
Varying Input	96	96	0.786	0.680
Varying Input	128	96	0.815	0.700
Varying Input	256	96	1.054	0.808
Varying Horizon	96	50	0.571	0.549
Varying Horizon	96	96	0.786	0.680
Varying Horizon	96	150	0.903	0.751
Varying Horizon	96	200	0.946	0.777

Supplementary Table 8. Comparing the efficiency of Delayformer and ARNN on the ETTh2 and Weather datasets.

Models		Ours		ARNN	
Metric		MSE	MAE	MSE	MAE
ETTh2	96	0.290	0.338	0.352	0.387
	192	0.369	0.391	0.427	0.429
	336	0.423	0.427	0.454	0.454
	720	0.427	0.441	0.450	0.460
	Avg	0.377	0.399	0.421	0.433
Weather	96	0.160	0.210	2.778	1.062
	192	0.208	0.252	2.926	1.104
	336	0.265	0.293	3.008	1.141
	720	0.346	0.346	3.210	1.217
	Avg	0.245	0.275	2.981	1.131

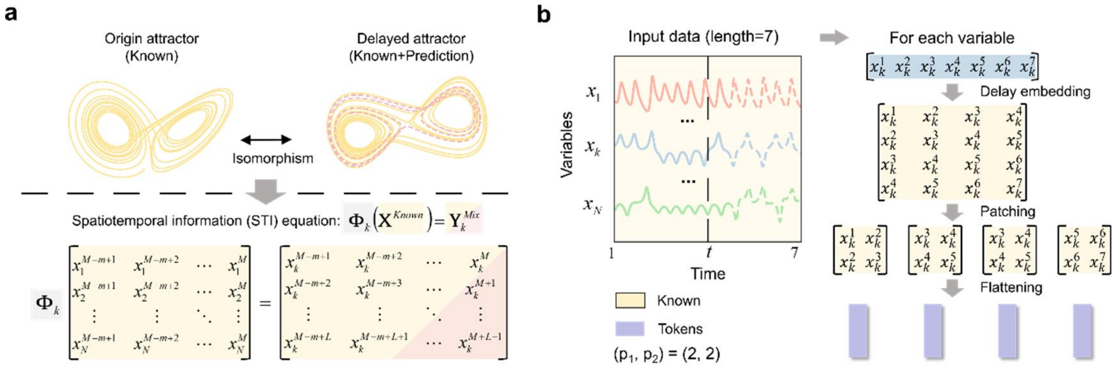

Supplementary Fig. 1. STI framework and example for token construction in Delayformer. (a) The STI equation based on the delayed embedding theorem can transform the spatial information of high-dimensional data ($\boldsymbol{X}^{Known} = \{\boldsymbol{X}^t\}_{t=M-m+1}^{M}$) to the temporal information ($\mathbf{Y}_k^{Mix} = \{\mathbf{Y}_k^t\}_{t=M-m+1}^{M}$, $\mathbf{Y}_k^t = (x_k^{t+1}, x_k^{t+2}, \dots, x_k^{t+L})^T$ with $L > 1$ as the embedding dimension) of any target variable. However, STI equation can only predict one variable at a time, which is extremely inefficient for high-dimensional data. **(b)** A time series data with length 7 and N variables are taken as an example for constructing tokens in Delayformer. The data of each variable is reconstructed into Hankel matrices by delay embedding. In the ViT encoder, Hankel matrices are sliced into patches and then flattened into tokens as the input of transformer encoder (Vanilla Transformer).

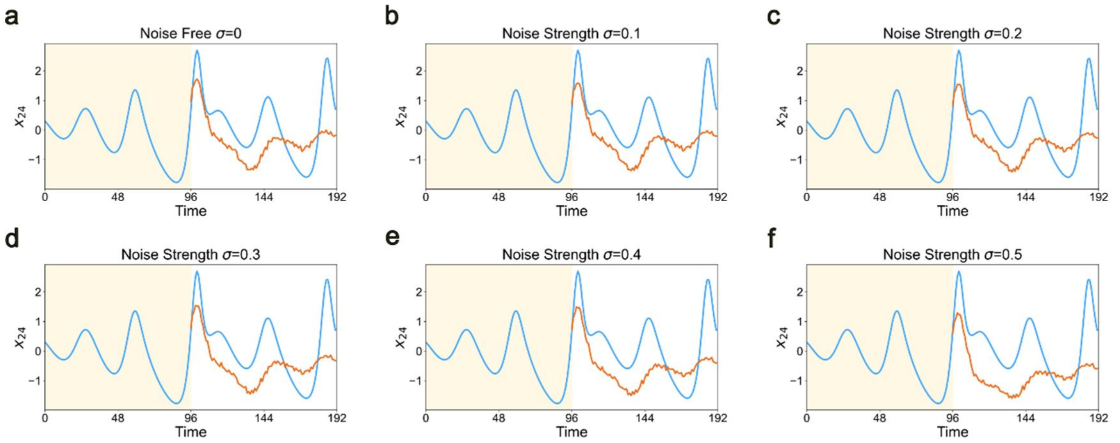

Supplementary Fig. 2. Validation of iTransformer on time series data of a 30D coupled Lorenz system. Future state predictions (blank regions) for a sliding window of input data (shaded region) in the test set, where the blue curve represents the ground truth, and the orange curve represents the iTransformer (corresponding to one gray curve in Fig.2). **(a)** Noise-free condition. **(b-f)** Conditions with additive Gaussian noise, mean value of 0, and noise strength σ=0.1,0.2,0.3,0.4,0.5.

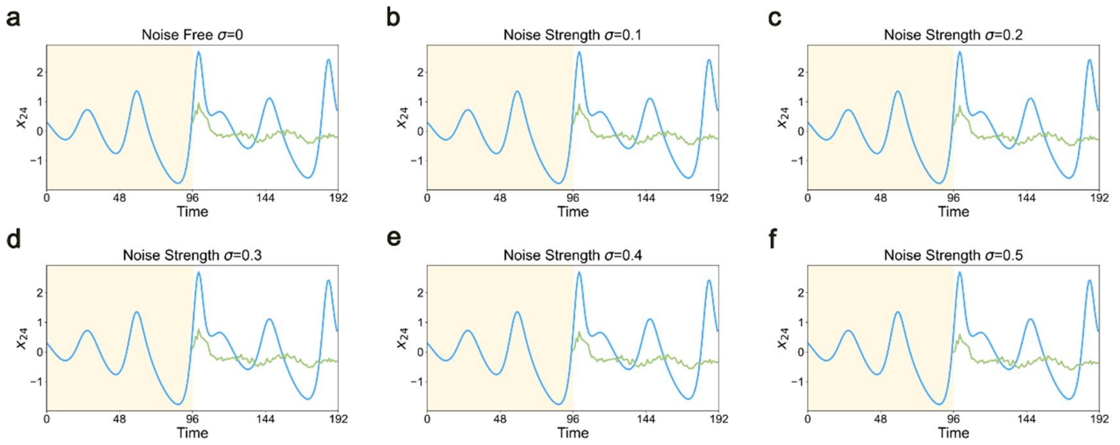

Supplementary Fig. 3. Validation of PatchTST on time series data of a 30D coupled Lorenz system. Future state predictions (blank regions) for a sliding window of input data (shaded region) in the test set, where the blue curve represents the ground truth, and the green curve represents the PatchTST (corresponding to one gray curve in Fig.2). **(a)** Noise-free condition. **(b-f)** Conditions with additive Gaussian noise, mean value of 0, and noise strength σ=0.1,0.2,0.3,0.4,0.5.

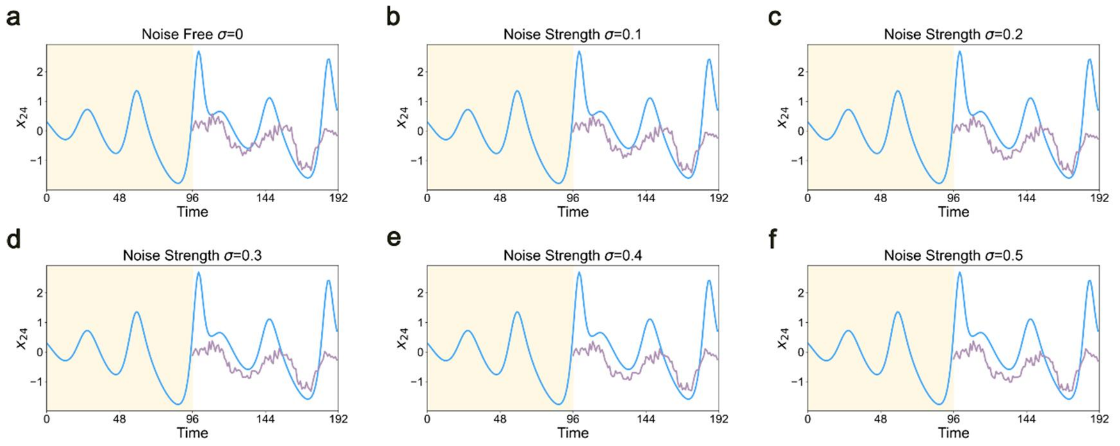

Supplementary Fig. 4. Validation of TimesNet on time series data of a 30D coupled Lorenz system. Future state predictions (blank regions) for a sliding window of input data (shaded region) in the test set, where the blue curve represents the ground truth, and the purple curve represents the TimesNet (corresponding to one gray curve in Fig.2). **(a)** Noise-free condition. **(b-f)** Conditions with additive Gaussian noise, mean value of 0, and noise strength σ=0.1,0.2,0.3,0.4,0.5.

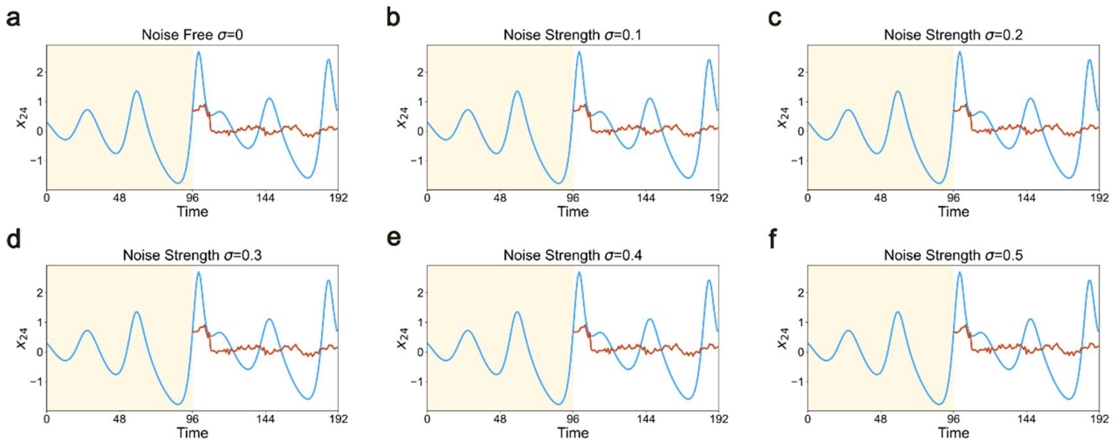

Supplementary Fig. 5. Validation of Crossformer on time series data of a 30D coupled Lorenz system. Future state predictions (blank regions) for a sliding window of input data (shaded region) in the test set, where the blue curve represents the ground truth, and the dark red curve represents the Crossformer (corresponding to one gray curve in Fig.2). **(a)** Noise-free condition. **(b-f)** Conditions with additive Gaussian noise, mean value of 0, and noise strength σ=0.1,0.2,0.3,0.4,0.5.

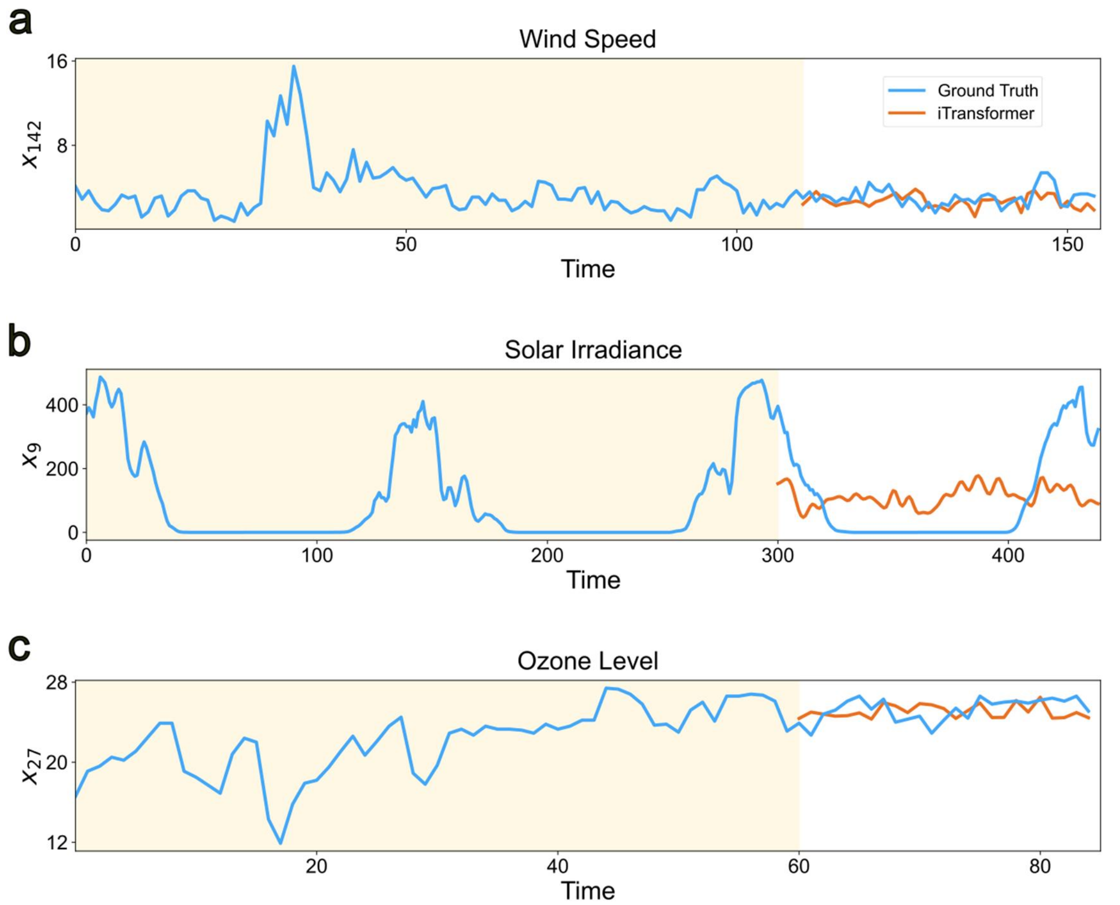

Supplementary Fig. 6. Predictions using iTransformer on three meteorological datasets. We applied iTransformer to three meteorological datasets with limited training data (corresponding to Fig. 3). Each

figure exhibits predictions of future states (blank region) based on the training data (shadow region), where the blue curve represents the ground truth, the orange curve represents the prediction of iTransformer (corresponding to one gray curve in Fig.3). **(a)** Predictions on the wind speed dataset (155 dimensions). The number of known data points is 110, and the predicted unknown data points is 45. **(b)** Predictions on the solar irradiance dataset (155 dimensions). The number of known data points is 300, and the predicted unknown data points is 140. **(c)** Predictions on the ground ozone-level dataset (72 dimensions). The number of known data points is 160, and the predicted unknown data points is 25.

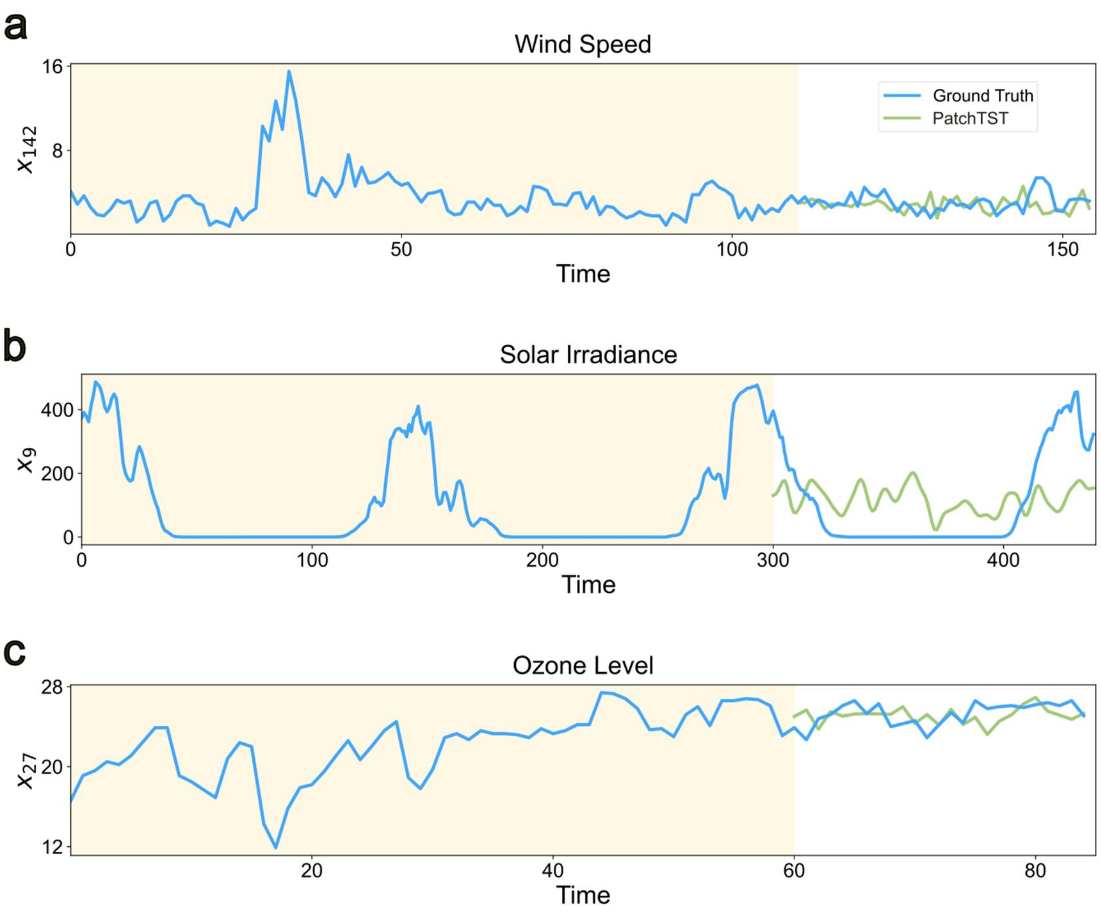

Supplementary Fig. 7. Predictions using PatchTST on three meteorological datasets. We applied iTransformer to three meteorological datasets with limited training data (corresponding to Fig. 3). Each figure exhibits predictions of future states (blank region) based on the training data (shadow region), where the blue curve represents the ground truth, the green curve represents the prediction of PatchTST (corresponding to one gray curve in Fig.3). **(a)** Predictions on the wind speed dataset (155 dimensions). The number of known data points is 110, and the predicted unknown data points is 45. **(b)** Predictions on the solar irradiance dataset (155 dimensions). The number of known data points is 300, and the predicted unknown data points is 140. **(c)** Predictions on the ground ozone-level dataset (72 dimensions). The number of known data points is 160, and the predicted unknown data points is 25.

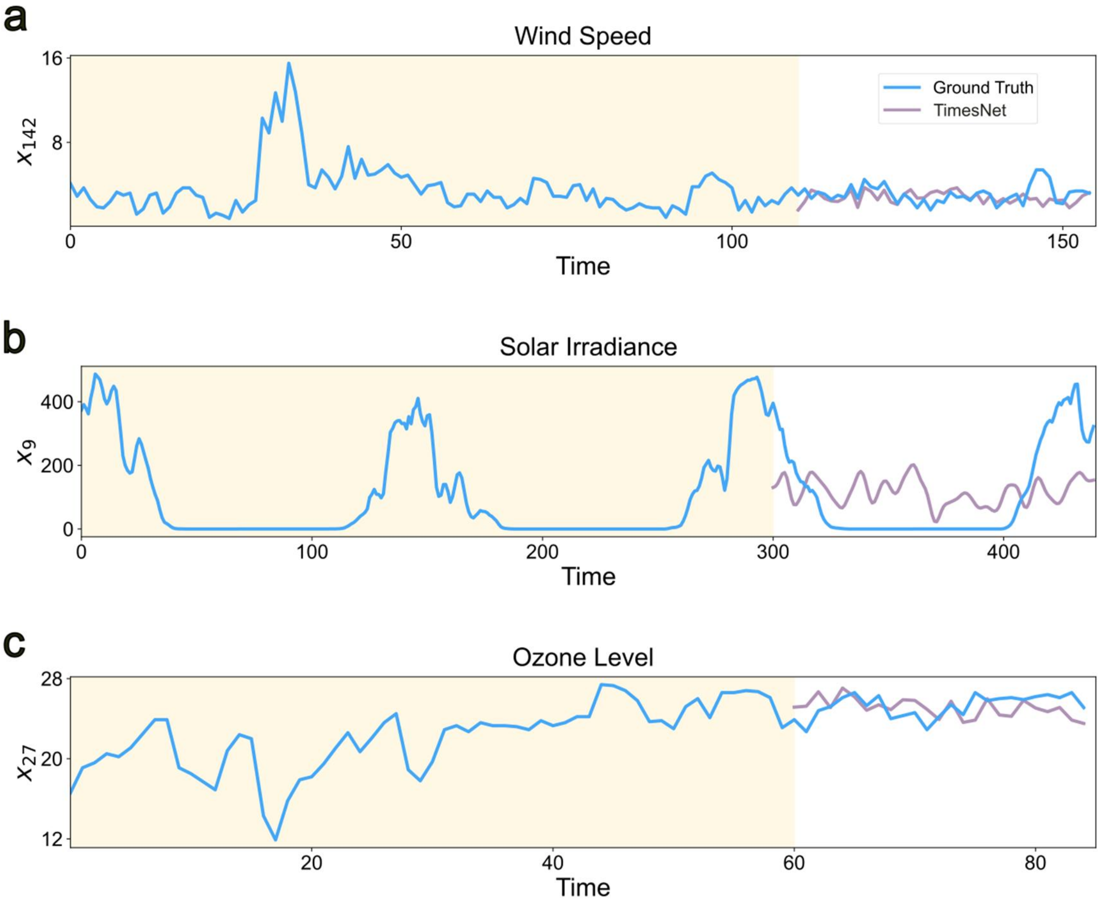

Supplementary Fig. 8. Predictions using TimesNet on three meteorological datasets. We applied iTransformer to three meteorological datasets with limited training data (corresponding to Fig. 3). Each figure exhibits predictions of future states (blank region) based on the training data (shadow region), where the blue curve represents the ground truth, the purple curve represents the prediction of TimesNet (corresponding to one gray curve in Fig.3). **(a)** Predictions on the wind speed dataset (155 dimensions). The number of known data points is 110, and the predicted unknown data points is 45. **(b)** Predictions on the solar irradiance dataset (155 dimensions). The number of known data points is 300, and the predicted unknown data points is 140. **(c)** Predictions on the ground ozone-level dataset (72 dimensions). The number of known data points is 160, and the predicted unknown data points is 25.

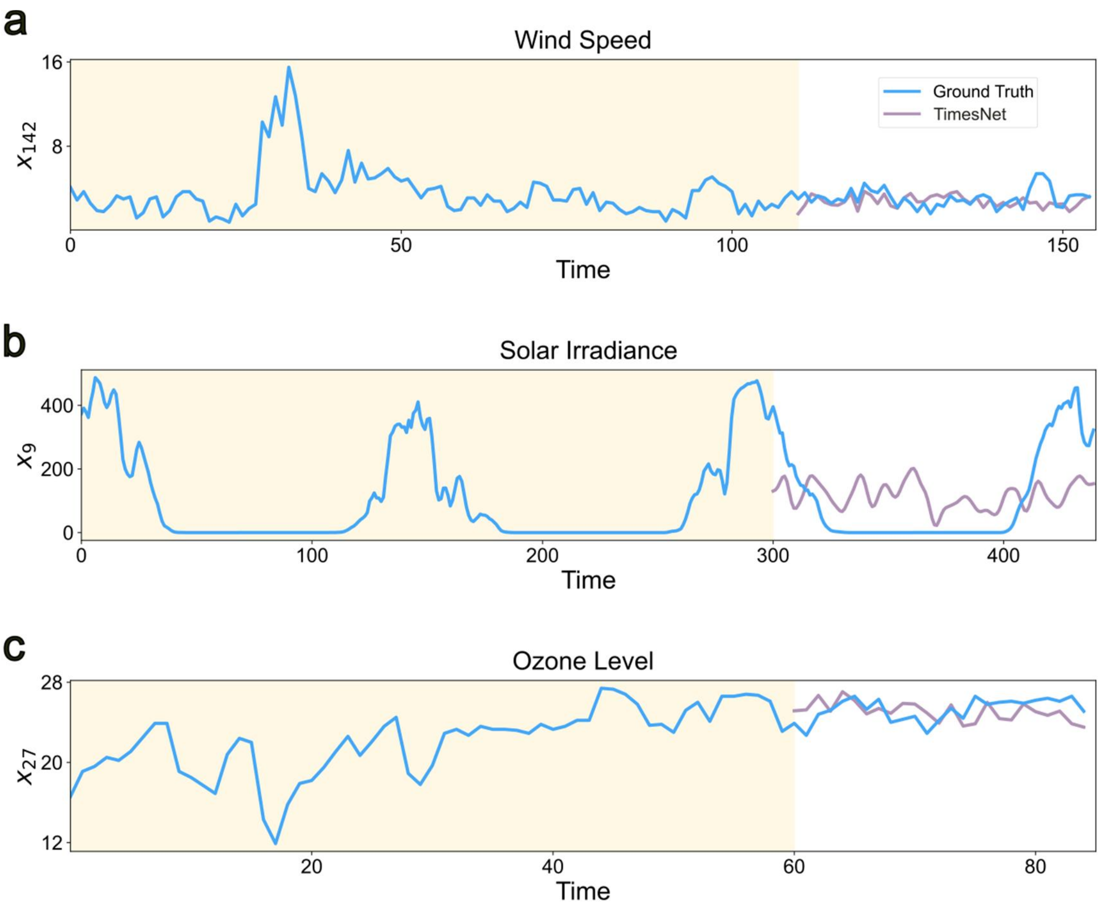

Supplementary Fig. 9. Predictions using Crossformer on three meteorological datasets. We applied iTransformer to three meteorological datasets with limited training data (corresponding to Fig. 3). Each figure exhibits predictions of future states (blank region) based on the training data (shadow region), where the blue curve represents the ground truth, the purple curve represents the prediction of Crossformer (corresponding to one gray curve in Fig.3). **(a)** Predictions on the wind speed dataset (155 dimensions). The number of known data points is 110, and the predicted unknown data points is 45. **(b)** Predictions on the solar irradiance dataset (155 dimensions). The number of known data points is 300, and the predicted unknown data points is 140. **(c)** Predictions on the ground ozone-level dataset (72 dimensions). The number of known data points is 160, and the predicted unknown data points is 25.

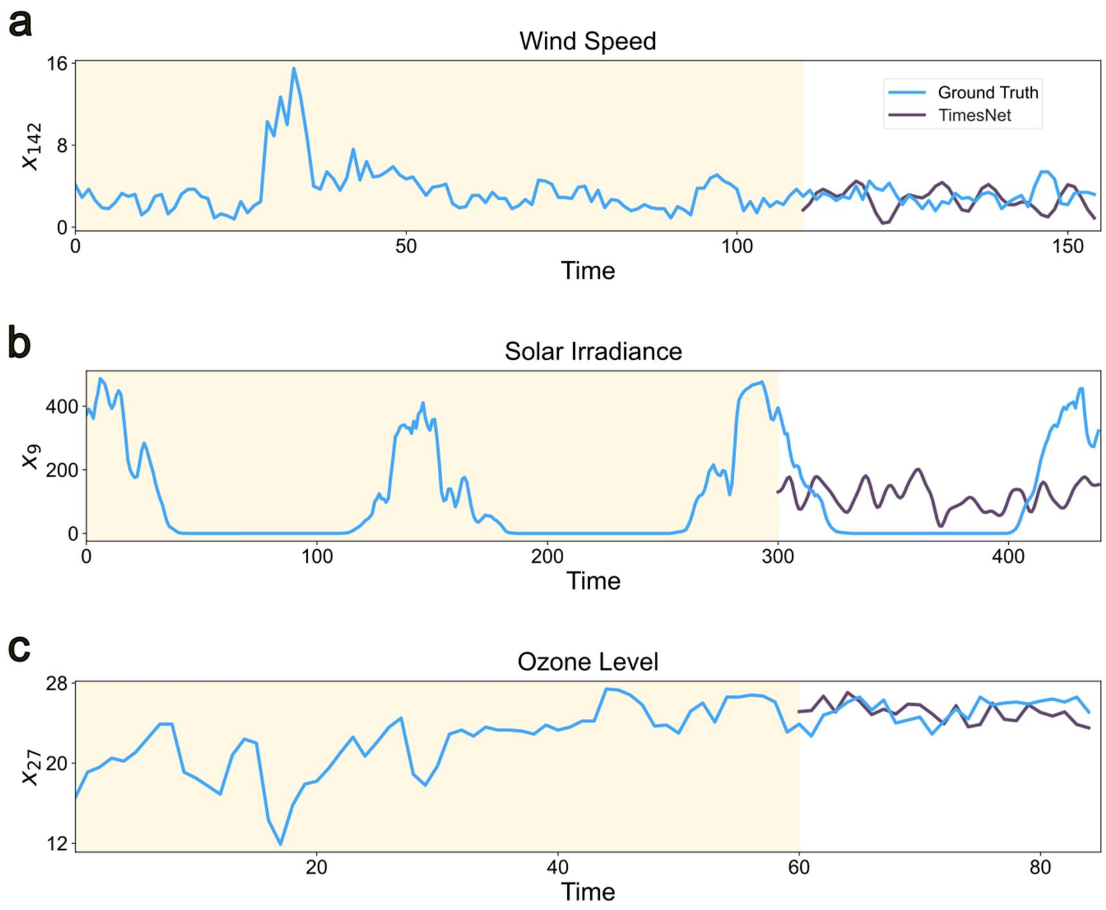

Supplementary Fig. 10. Predictions using Transformer on three meteorological datasets. We applied iTransformer to three meteorological datasets with limited training data (corresponding to Fig. 3). Each figure exhibits predictions of future states (blank region) based on the training data (shadow region), where the blue curve represents the ground truth, the dark purple curve represents the prediction of Transformer (corresponding to one gray curve in Fig.3). **(a)** Predictions on the wind speed dataset (155 dimensions). The number of known data points is 110, and the predicted unknown data points is 45. **(b)** Predictions on the solar irradiance dataset (155 dimensions). The number of known data points is 300, and the predicted unknown data points is 140. **(c)** Predictions on the ground ozone-level dataset (72 dimensions). The number of known data points is 160, and the predicted unknown data points is 25.

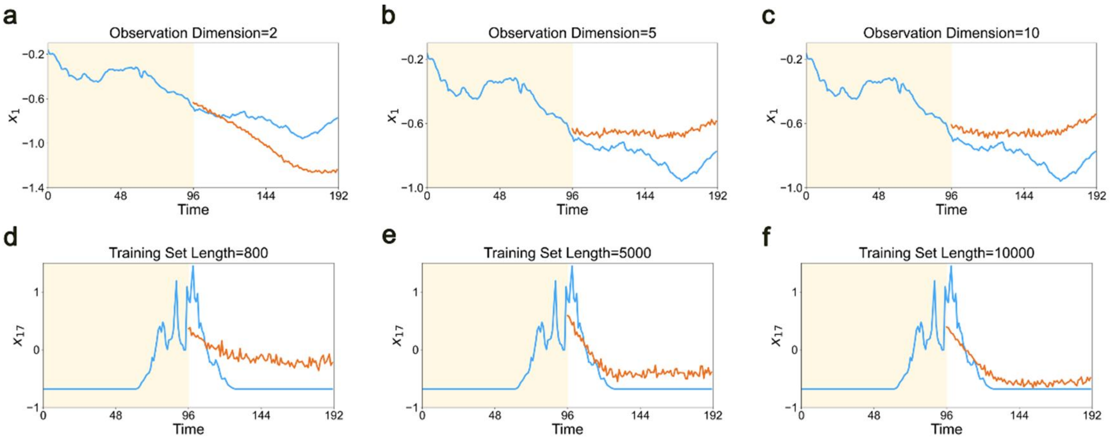

Supplementary Fig. 11. Performance of iTransformer under varying training set sizes and observation dimensions (corresponding to one gray curve in Fig.4). (a-c) Predictions of future states with different numbers of observed variables: **(a)** 2 variables, **(b)** 5 variables, **(c)** 10 variables. The shaded region represents one sliding window of input data, with the blue curve indicating the ground truth, the red curve showing Delayformer predictions, and the gray curves depicting baseline model predictions in the blank region. **(d-f)** Predictions of future states (unshaded region) using different training set sizes: **(d)** 800 points, **(e)** 5,000 points, **(f)** 10,000 points. Again, the shaded region represents one sliding window of input data, with the blue curve indicating the ground truth, the red curve showing Delayformer predictions, and the gray curves depicting baseline model predictions in the blank region.

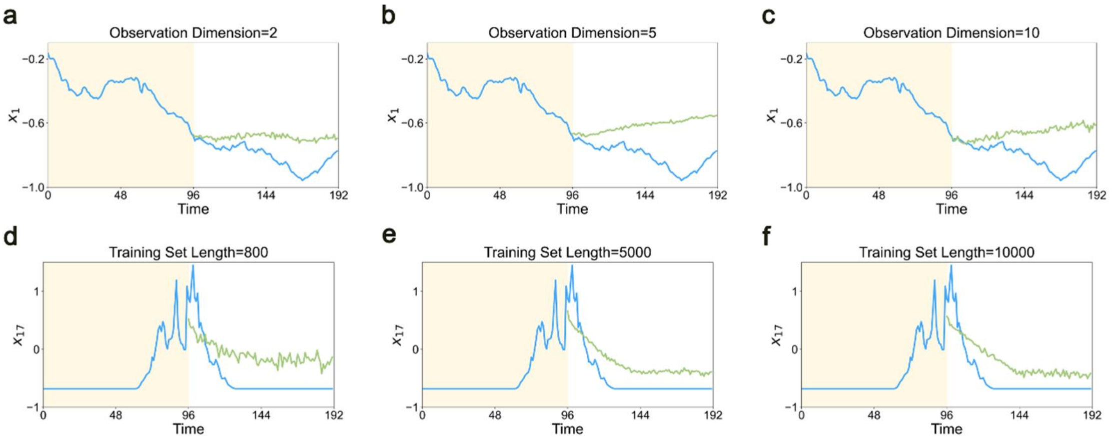

Supplementary Fig. 12. Performance of PatchTST under varying training set sizes and observation dimensions (corresponding to one gray curve in Fig.4). (a-c) Predictions of future states with different numbers of observed variables: **(a)** 2 variables, **(b)** 5 variables, **(c)** 10 variables. The shaded region represents one sliding window of input data, with the blue curve indicating the ground truth, the red curve showing Delayformer predictions, and the gray curves depicting baseline model predictions in the blank region. **(d-f)** Predictions of future states (unshaded region) using different training set sizes: **(d)** 800 points, **(e)** 5,000 points, **(f)** 10,000 points. Again, the shaded region represents one sliding window of input data, with the blue curve indicating the ground truth, the red curve showing Delayformer

predictions, and the gray curves depicting baseline model predictions in the blank region.

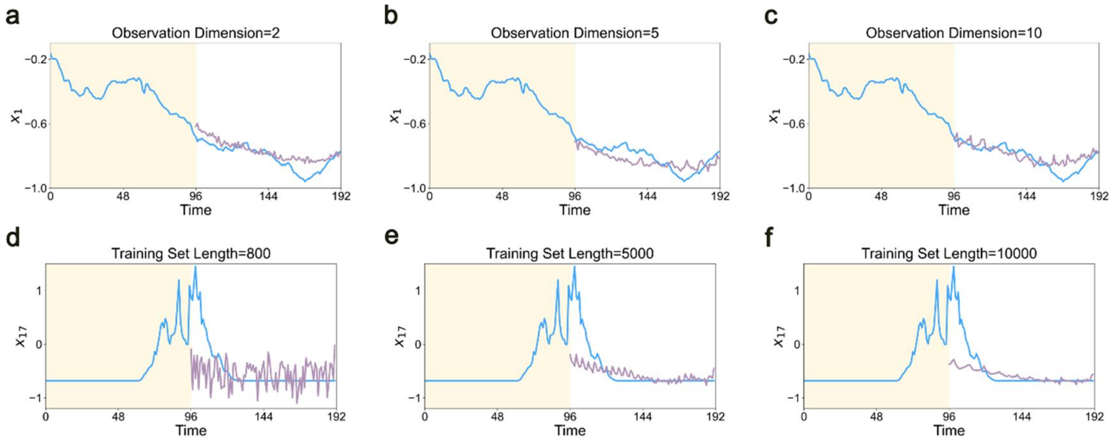

Supplementary Fig. 13. Performance of TimesNet under varying training set sizes and observation dimensions (corresponding to one gray curve in Fig.4). (a-c) Predictions of future states with different numbers of observed variables: **(a)** 2 variables, **(b)** 5 variables, **(c)** 10 variables. The shaded region represents one sliding window of input data, with the blue curve indicating the ground truth, the red curve showing Delayformer predictions, and the gray curves depicting baseline model predictions in the blank region. **(d-f)** Predictions of future states (unshaded region) using different training set sizes: **(d)** 800 points, **(e)** 5,000 points, **(f)** 10,000 points. Again, the shaded region represents one sliding window of input data, with the blue curve indicating the ground truth, the red curve showing Delayformer predictions, and the gray curves depicting baseline model predictions in the blank region.

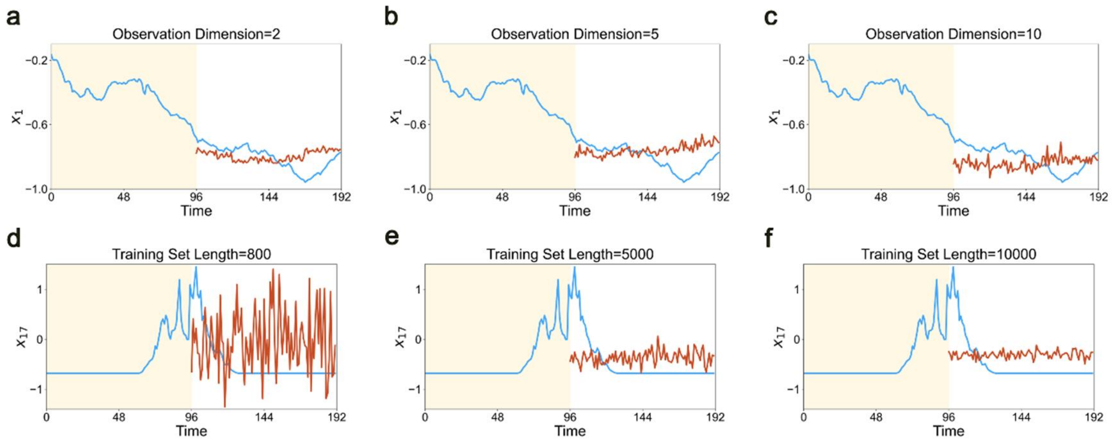

Supplementary Fig. 14. Performance of Crossformer under varying training set sizes and observation dimensions (corresponding to one gray curve in Fig.4). (a-c) Predictions of future states with different numbers of observed variables: **(a)** 2 variables, **(b)** 5 variables, **(c)** 10 variables. The shaded region represents one sliding window of input data, with the blue curve indicating the ground truth, the red curve showing Delayformer predictions, and the gray curves depicting baseline model predictions in

the blank region. **(d-f)** Predictions of future states (unshaded region) using different training set sizes: **(d)** 800 points, **(e)** 5,000 points, **(f)** 10,000 points. Again, the shaded region represents one sliding window of input data, with the blue curve indicating the ground truth, the red curve showing Delayformer predictions, and the gray curves depicting baseline model predictions in the blank region.

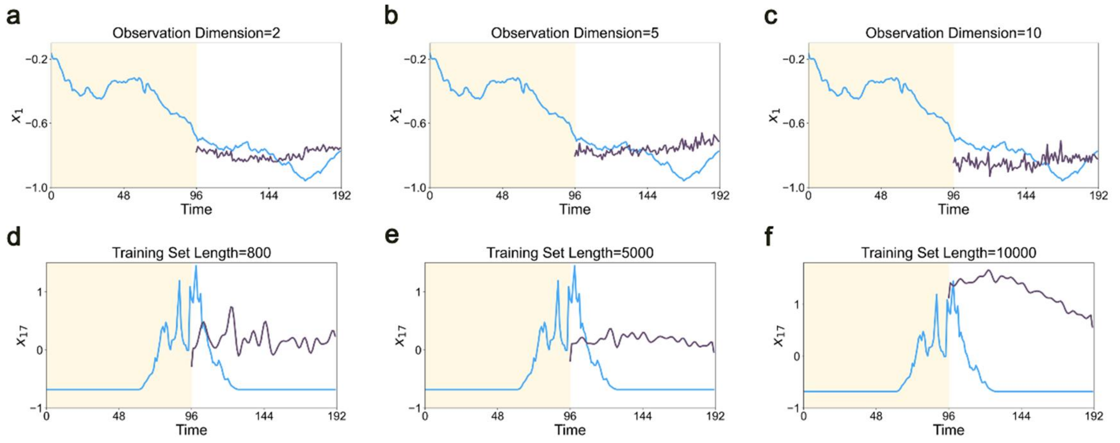

Supplementary Fig. 15. Performance of Transformer under varying training set sizes and observation dimensions (corresponding to one gray curve in Fig.4). (a-c) Predictions of future states with different numbers of observed variables: **(a)** 2 variables, **(b)** 5 variables, **(c)** 10 variables. The shaded region represents one sliding window of input data, with the blue curve indicating the ground truth, the red curve showing Delayformer predictions, and the gray curves depicting baseline model predictions in the blank region. **(d-f)** Predictions of future states (unshaded region) using different training set sizes: **(d)** 800 points, **(e)** 5,000 points, **(f)** 10,000 points. Again, the shaded region represents one sliding window of input data, with the blue curve indicating the ground truth, the red curve showing Delayformer predictions, and the gray curves depicting baseline model predictions in the blank region.

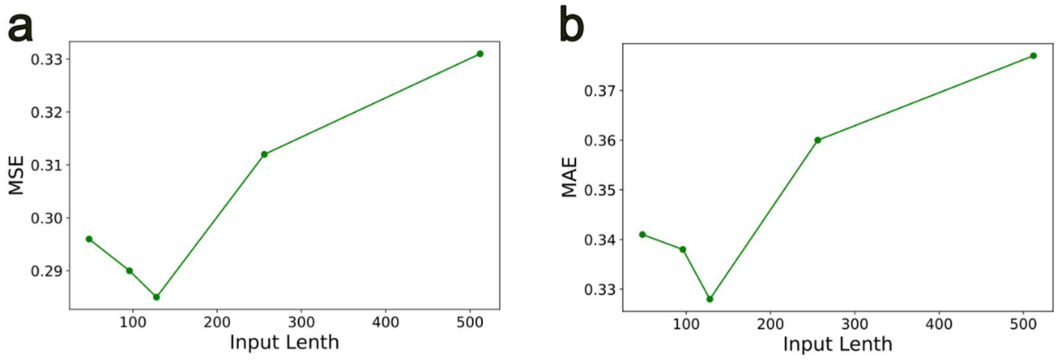

Supplementary Fig. 16. Performance of Delayformer on the ETTh2 dataset with varying input sequence lengths. The figure displays the Mean Squared Error (MSE) on the test set for a 96-step prediction task. The model was trained and evaluated with five different input sequence lengths: 48, 96,

128, 256, and 512. The results indicate that performance generally improves as the input length increases, with the optimal performance observed at an input length of 128. A slight increase in error at an input length of 512 suggests that overly long historical sequences can introduce noise, demonstrating a trade-off between information content and relevance.

Supplementary References

1. Nie Y, Nguyen NH, Sinthong P *et al.* A time series is worth 64 words: Long-term forecasting with transformers. *arXiv preprint arXiv:221114730*. 2022.
2. Zhang Y, Yan J. Crossformer: Transformer utilizing cross-dimension dependency for multivariate time series forecasting. In: *The eleventh international conference on learning representations, 2023*.
3. Zhou T, Ma Z, Wen Q *et al.* Fedformer: Frequency enhanced decomposed transformer for long-term series forecasting. In: *International conference on machine learning, 2022*, p. 27268-27286. PMLR.
4. Liu Y, Wu H, Wang J *et al.* Non-stationary transformers: Exploring the stationarity in time series forecasting. *Advances in Neural Information Processing Systems*. 2022; **35**: 9881-9893.
5. Wu H, Xu J, Wang J *et al.* Autoformer: Decomposition transformers with auto-correlation for long-term series forecasting. *Advances in neural information processing systems*. 2021; **34**: 22419-22430.
6. Wu H, Hu T, Liu Y *et al.* Timesnet: Temporal 2d-variation modeling for general time series analysis. *arXiv preprint arXiv:221002186*. 2022.
7. Li Z, Qi S, Li Y *et al.* Revisiting long-term time series forecasting: An investigation on linear mapping. *arXiv preprint arXiv:230510721*. 2023.
8. Zeng A, Chen M, Zhang L *et al.* Are transformers effective for time series forecasting? In: *Proceedings of the AAAI conference on artificial intelligence, 2023*. Abstract 9, p. 11121-11128.
